# The Multifaceted Role of Extracellular Vesicles in Triple Negative Breast Cancer

**DOI:** 10.3390/ijms27135976

**Published:** 2026-07-03

**Authors:** Serena El Rayes, Ebaa Ababneh, Varun Nannuri, Manjusha Vaidya, Kiminobu Sugaya, Jihe Zhao

**Affiliations:** Burnett School of Biomedical Sciences, College of Medicine, University of Central Florida, 6900 Lake Nona Boulevard, Orlando, FL 32827, USA

**Keywords:** extracellular vesicle, triple negative breast cancer

## Abstract

Triple negative breast cancer (TNBC) is an aggressive and heterogeneous subtype of breast cancer characterized by the absence of the estrogen receptor (ER), progesterone receptor (PR), and human epidermal growth factor receptor 2 (HER2), resulting in limited options for targeted therapy and high rates of metastasis, recurrence and death. Extracellular vesicles (EVs) have emerged as central mediators of TNBC pathophysiology, functioning as key intercellular communication vehicles transporting oncogenic proteins, nucleic acids, lipids, and metabolites. These EV-mediated interactions promote tumor microenvironment (TME) remodeling, immune evasion, metastatic niche formation, and therapeutic resistance. Given their stability, accessibility, and molecular complexity, EVs also represent promising diagnostic and prognostic biomarkers for TNBC. Advances in isolation and molecular profiling technologies have enabled the identification of EV-associated signatures that predict therapeutic response and stratify patient risk. Beyond their utility as biomarkers, EVs are rapidly emerging as therapeutic targets and delivery platforms, demonstrating efficacy in transporting chemotherapeutics, RNA-based therapeutics, immune modulators, and photosensitizers with enhanced targeting specificity and therapeutic efficiency. Collectively, EVs play a multifaceted role in TNBC biology, serving simultaneously as drivers of disease progression, minimally invasive biomarkers, and versatile therapeutic vehicles. The integration of EV-centered diagnostics, multi-omic profiling, and engineered therapeutics holds significant potential to transform TNBC management and advance precision oncology for this challenging breast cancer subtype.

## 1. Introduction

Extracellular vesicles (EVs) are membrane-bound nanoparticles released by the cells that play an important role in intercellular communication by transporting diverse cargo, including proteins, lipids, and nucleic acids. The uptake of EVs influences recipient cell behavior through various signaling mechanisms, including autocrine, paracrine, endocrine, and juxtacrine pathways, in both physiological and pathological contexts [1]. EVs can be classified into different types based on their size and biogenesis. Exosomes typically range from 30 to 150 nm in diameter and originate from the endosomal pathway, whereas microvesicles are larger EVs, ranging from 1 to 5 µm in diameter, and are generated through outward budding of the plasma membrane. Apoptotic bodies, in contrast, are particularly released during programmed cell death. However, because vesicle biogenesis is often not experimentally confirmed, the International Society for Extracellular Vesicles (ISEV) recommends the use of the generic term “EV” or operational terms such as small EVs (sEVs) unless endosomal origin is directly demonstrated, in accordance with the updated MISEV 2023 guidelines [2]. Hence, vesicles previously described as “exosomes” based primarily on size or marker enrichment are herein referred to as EVs as well.

In cancer biology, EVs have emerged as key modulators of tumor progression, immune evasion, angiogenesis, metastasis, and therapeutic resistance. Cancer-associated EVs can carry and deliver oncogenic molecules to the tumor microenvironment (TME), thereby promoting a pro-tumoral, immunosuppressive milieu. For example, elevated levels of cancer-specific miRNAs in serum-derived EVs that accumulate in the liver of gastric cancer patients with liver metastasis have been shown to activate signaling pathways that promote angiogenesis, immune suppression, metastasis, and poor prognosis [3]. Similarly, in non-small cell lung cancer, EV-mediated transfer of circular RNA (circVMP1) has been shown to promote tumor progression, chemoresistance, cell survival and immune evasion [4]. Due to their small size, targeting specificity, and efficient cargo transport capability, EVs have also emerged as promising tools in anticancer therapy, serving as delivery vehicles for bioactive molecules such as mRNAs, miRNAs, proteins, and lipids to selectively target cancer cells [5]. For example, it has been shown that the large-scale production of therapeutic EVs loaded with miRNA inhibitors and chemotherapeutic agents can effectively reverse drug resistance in colon cancer cells [6].

Triple-negative breast cancer (TNBC) is an aggressive, heterogeneous subtype of breast cancer (BC) that is predominantly diagnosed in young premenopausal women [7]. Constituting 10–20% of all BC cases, TNBC lacks expression of the estrogen receptor (ER), progesterone receptor (PR), and human epidermal growth factor 2 (HER2) [8]. This prevents the possibility of the hormonal or anti-HER2 targeted therapies widely used for other BC subtypes and restricts treatment options primarily to chemotherapy. However, approximately 50% of TNBC patients eventually develop chemoresistance [9]. Therefore, developing strategies for early detection, accurate prognosis, and innovative therapeutic intervention is critical for improving patient outcomes.

Recent studies have highlighted the intricate interplay between EVs and TNBC, revealing that TNBC-derived EVs contribute significantly to tumor aggressiveness and immune evasion by carrying oncogenic cargo that modulates the TME and promotes angiogenesis [10,11]. Given their molecular enrichment and stability in circulation, EVs hold considerable promise in TNBC diagnosis, therapy, and prognosis. The isolation and analysis of distinct EV subpopulations facilitate advanced cancer diagnostics and personalized treatment strategies, enabling discrimination between TNBC and other BC subtypes [12]. At the prognostic level, EV cargo such as miRNA correlates with tumor grade, immunotherapy resistance, T cell function, and recurrence risk [11]. Additionally, engineered EVs can deliver therapeutic cargo including siRNAs, chemotherapeutic agents and immune modulators directly and specifically to TNBC cells to enhance cell apoptosis, suppress proliferation, and minimize metastatic potential [13].

Despite the clinical urgency of TNBC and the translational promise of EV-based technologies, a comprehensive synthesis of the intersection between EV biology and TNBC biology remains lacking. This review aims to consolidate current knowledge of EV biology in TNBC, with particular focus on emerging biomarkers and therapeutic strategies. By bridging molecular insights with clinical application and advanced therapeutic innovation, we aim to advance EV-based approaches for the diagnosis and treatment of TNBC. Importantly, this review also highlights the opportunities and challenges associated with translating EV-based biomarkers and therapeutics into clinical practice.

## 2. Role of EVs in TNBC Pathophysiology

**Figure 1:**
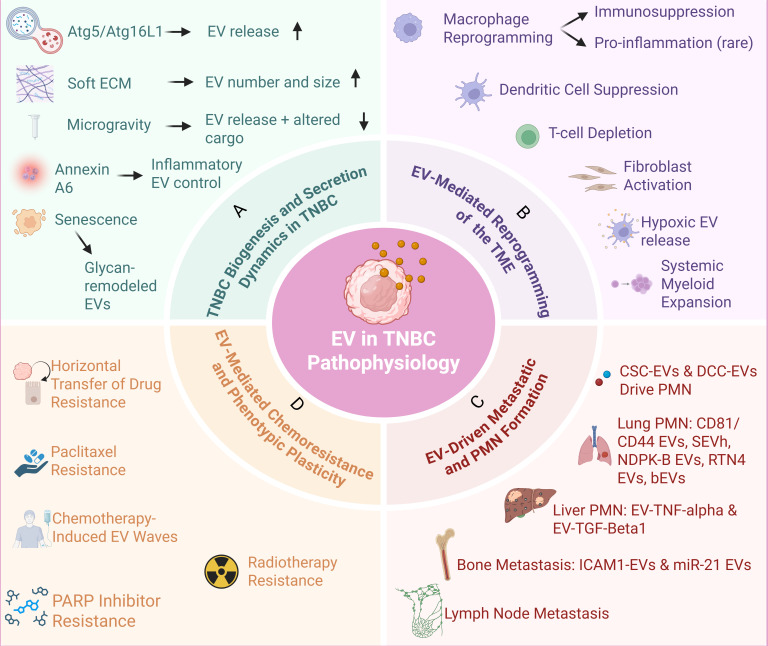
**EV-mediated regulation of pathology in TNBC.** (**A**) Regulation of EV biogenesis and secretion dynamics. (**B**) EV-mediated reprogramming of the tumor microenvironment, including immune suppression and stromal activation. (**C**) EV-driven metastasis and pre-metastatic niche formation. (**D**) EV-mediated therapeutic resistance and related phenotypic plasticity. Abbreviations: Atg5, autophagy-related 5; Atg16L1, autophagy-related 16-like 1; CSC-EVs, cancer stem cell–derived extracellular vesicles; DCC-EVs, differentiated cancer cell-derived extracellular vesicles; ECM, extracellular matrix; EVs, extracellular vesicles; ICAM-1, intercellular adhesion molecule 1; miR-21, microRNA-21; NDPK-B, nucleoside diphosphate kinase B; PARP, poly(ADP-ribose) polymerase; PMN, pre-metastatic niche; RTN4, reticulon 4; TGF-β1, transforming growth factor beta 1; TNBC, triple-negative breast cancer; TNF-α, tumor necrosis factor alpha; TME, tumor microenvironment.

Before discussing the emerging diagnostic, prognostic, and therapeutic applications of EVs in TNBC, it is important to understand their biological foundation that underlies these clinical utilities. EVs function as biomarkers and therapeutic targets due to their central role in TNBC pathophysiology. TNBC cells exploit EVs as high-capacity intercellular communication vectors that actively remodel the TME, coordinate metastasis, enhance tumor growth, and promote other aggressive phenotypes. Through the EV-mediated transfer of pathogenic molecular cargo, including miRNAs, proteins, lipids, and metabolites, TNBC cells enhance stemness, induce chemoresistance, and reprogram the tumor stromal cells including immune cells [14]. Importantly, TNBC cells release both a greater quantity of EVs and more functionally disruptive cargo than other BC subtypes, thereby amplifying their impact on tumor behavior. Figure 1 summarizes the mechanisms through which EV signaling shapes TNBC progression, immune remodeling, metastasis, and therapeutic resistance.

### 2.1. EV Biogenesis and Secretion Dynamics in TNBC

A defining characteristic of TNBC is its unusually high EV output, which underlies many of its downstream pathogenic processes. However, the upstream mechanisms underlying this heightened vesicle production remains poorly understood. Recent studies have revealed that autophagy-related proteins, particularly Atg5, can potentiate metastasis by increasing EV output through a pathway independent of canonical macro-autophagy [15]. Specifically, loss of Atg5 or Atg16L1 markedly reduces EV secretion; whereas, Atg7 deficiency has no effect [15]. This demonstrates that the mechanism is distinct from classical autophagy [15]. Mechanistically, Atg5 interacts with LC3 to disrupt the V1V0-ATPase by removing the regulatory subunit ATP6V1E1, thereby preventing proper acidification of late endosomes and multivesicular bodies (MVBs) [15]. This impaired acidification favors MVB maturation toward exosome release rather than lysosomal degradation [15]. Beyond intracellular regulators such as Atg5, the physical properties of the TME also influence EV biogenesis. Substrate stiffness has emerged as a key regulator of EV secretion in TNBC cells where softer extracellular matrix (ECM) induces cell softening, rounding, cytoskeletal relaxation, and a marked increase in the number and size of released sEVs in the TME [16]. Thus, ECM softness enhances larger EV output and contributes to the overall elevated vesicle burden characteristic of metastatic TNBC [16]. Furthermore, microgravity has been shown to impose a distinct biophysical stress that reshapes EV biogenesis and cargo composition in TNBC cells. Cells exposed to stimulated microgravity exhibit reduced EV release, increased EV size, and a less aggressive phenotype mediated through EV-encoded signaling alterations [17]. Additionally, annexin A6 has recently been shown to regulate both pro-inflammatory cytokine secretion and seral EV (sEV) release through its interaction with the vesicle fusion SNARE protein SNAP23 [18]. Downregulation of annexin A6 markedly reduces the secretion of MCP-1, IL-1, DKK1, TSP-1, and osteopontin, while simultaneously suppressing sEV output and decreasing EV-associated Rab7 and cholesterol content, positioning annexin A6 as a key coordinator of inflammatory EV output in TNBC [18]. Furthermore, TNBC cells can dynamically remodel the molecular composition of their EVs in response to therapeutic stress. Therapy-induced senescence (TIS), for example, profoundly alters the N-glycan landscape of TNBC cells and their EV progeny, indicating that senescence reprograms both intracellular glycosylation pathways and the glycan composition of secreted vesicles [19]. Correspondingly, lectin-binding proteins, including a lower-molecular-weight calnexin species and proteolytically cleaved galectin-3, are significantly altered during senescence [19].

Importantly, in TNBC, EV-mediated intercellular communication in TNBC appears to rely on the full spectrum of vesicle sub-populations rather than a single pathogenic fraction [20]. Studies across three TNBC cell lines have demonstrated that pharmacologic inhibition of EV biogenesis pathways via calpeptin, Y27632, manumycin A, GW4869, or their combinations, reduces EV release by 64–98%, indicating that TNBC cells depend on multiple, heterogeneous, and partially redundant mechanisms [20]. Importantly, even the small fraction of EVs that remained biologically active after inhibition, albeit with diminished potency, can still transmit pro-migratory, aggressive phenotypes to recipient cells [20]. These findings underscore that all EV subtypes contribute to TNBC’s pathogenic signaling, and that partial suppression of EV release is insufficient to fully block the transfer of malignant traits [20].

### 2.2. EV-Driven Cargo Loading Mechanisms

TNBC cells do not package EV cargo randomly. Instead, they use regulated, pathway-specific sorting mechanisms that preferentially enrich oncogenic miRNAs, proteins, and lipids. These mechanisms create EV populations that encode an invasive, immunosuppressive, and pro-metastatic phenotype.

First, selective cargo loading is influenced by membrane-topology determinants. It has been shown that CD133, a pentaspan transmembrane protein localized to membrane protrusions and lipid rafts, is associated with EV biogenesis, membrane organization, and selective cargo loading through the incorporation of pro-angiogenic factor CD105 [21]. CD133-positive EVs strongly promote endothelial tubulogenesis and early EV-mediated signaling specificity [21]. CSC-derived EVs also exhibit selective membrane-protein loading, exemplified by the enrichment of TSPAN8, an integral membrane protein, on their surface [22]. TSPAN8 protein functions as a topology-dependent sorting determinant that enables EVs to engage CD103 on T cells, triggering LKB1-AMPK signaling pathway and driving expansion of immunosuppressive Tregs [22]. TNBC EVs also display selective enrichment of surface CSF-1 together with luminal activators of cGAS-STING, forming a cargo signature that drives monocyte differentiation into pro-inflammatory, interferon-responsive macrophages [23]. This CSF-1-bearing EV subset is detectable within patient TAMs and correlates with increased T cell infiltration and improved clinical outcome, underscoring how specific surface-cargo configurations can imprint distinct immune-modulatory phenotypes [23].

Building on membrane-topology mechanisms, stress and cytokine-driven pathways also influence EV cargo loading. TNBC cells engage in cytokine-driven cargo selection through the selective enrichment of CXCL1 in paclitaxel-induced EVs [24]. CXCL1 loading is controlled by a stress-induced CXCL1–Myc–RAB31/FLOT2 axis that enhances intraluminal vesicle biogenesis, generating EVs that potently activate PD-L1-dependent M2 macrophage polarization and promote chemoresistance and metastatic progression [24]. Annexin A2 has emerged as a tumor-intrinsic determinant that reshapes the EV proteome in TNBC. AnxA2 expression drives the selective enrichment of cytoskeletal, adhesion, and trafficking proteins within EVs [25]. This allows enhanced uptake by lung fibroblasts and greater capacity to induce fibroblast activation and motility [25]. This highlights how tumor-cell scaffolding proteins can imprint a pro-metastatic proteomic signature into EVs. Tumor-cell CCL5 expression has been shown to regulate EV biogenesis, secretion, and cargo composition, generating EVs that program macrophages into a more pro-metastatic, cytokine secreting phenotype [26]. These CCL5-dependent EVs drive macrophage release of CXCL1, CTLA-4, IFNG, OPN, HGF, TGFB, and CCL19, illustrating how chemokine signaling upstream of MVB formation can imprint a potent immune-modulatory cargo signature [26]. This positions CCL5 as a tumor-intrinsic regulator of EV-mediated macrophage education and metastatic progression.

Functional cargo signatures can help reflect regulated sorting programs. Pro-aggregatory proteins such as UPAR and PDGFRβ have been shown to be enriched in EVs and accelerate platelet activation, linking specific cargo signatures to the thrombotic and pro-metastatic phenotype characteristic of aggressive TNBC [27]. SMR peptide treatment was shown to selectively enrich a cohort of tumor-suppressive miRNAs within TNBC-EVs which reduce inflammasome signaling and diminished IL-1β secretion, illustrating how targeted perturbations can redirect EV miRNA sorting toward anti-inflammatory and anti-metastatic profiles [28]. Further supporting the concept that EV miRNA composition is dynamically rewired by signaling cues, IL-3Rα blockade on tumor-derived endothelial cells selectively depletes miR-24-3p from TEC-EVs, generating a reprogrammed vesicle population that upregulates SPRY2 in TNBC cells and suppresses epithelial-to-mesenchymal transition (EMT), survival, and metastasis [29]. This IL-3Rα-dependent miRNA remodeling demonstrates that endothelial signaling pathways can imprint potent anti-metastatic instructions onto EV cargo, reinforcing the idea that EV miRNA loading is a regulated, therapeutically targetable process [29].

### 2.3. EV-Mediated Reprogramming of the TME

TNBC-derived EVs frequently promote metastatic and immunosuppressive programs to support tumor progression. In contrast, it has been shown that a subset of EV-mediated signaling pathways can instead induce pro-inflammatory, anti-tumor macrophage states associated with favorable clinical outcomes [23]. TNBC-derived EVs have been shown to induce macrophages with a robust interferon-stimulated gene signature. This is driven by the combined action of EV-surface CSF-1, which supports monocyte survival, and EV-associated cargo that activates cGAS-STING and related innate immune signaling pathways [23]. This transcriptional signature correlates with increased T cell infiltration and improved overall survival in TNBC cohorts [23]. On the other hand, EVs can also promote immunosuppressive remodeling, particularly in therapy-resistant settings, representing the more widely observed mechanism of TNBC-derived EV activity [30]. TNBC cells that survive the anti-PD-1 therapy and chemotherapy accumulate neutral lipids and enrich their EVs with arachidonic acid (AA), which reprograms tumor-associated neutrophils into a lipid-laden suppressive phenotype. This process results in upregulation of PGE2 and PD-L1, inhibition of CD8+ T cell activity, and reinforcement of immune evasion and treatment resistance [30]. Extending this lipid-driven immunosuppressive axis, cancer-associated adipocytes (CAAs) have also been recently shown to amplify immune evasion by upregulating membrane-bound PD-L1 and selectively enriching PD-L1 within tumor-derived EVs, thereby diminishing the efficacy of anti-PD-L1 therapy [31].

Beyond EV-mediated polarization of macrophages and neutrophils, TNBC cells have also been shown to directly impair dendritic cell function. Tumor-derived EVs (TDEVs) deliver CDC37 into conventional type 1 dendritic cell (cDC1) endosomes, where it sequesters antigens within HSP90 complexes and prevents cross-presentation, thereby blocking CD8+ T cell priming and promoting resistance to immune checkpoint blockade resistance [32]. Additionally, TNBC-derived EVs can exert measurable immunomodulatory effects on peripheral immune cells, reshaping natural killer (NK) and regulatory T (Treg) cell populations through a hyperactivation-associated depletion [33]. Altered activation of peripheral blood mononuclear cell (PBMC)-derived NK cells is characterized by increased CD333^+^/CD11b^+^ accompanied by a decrease in total NK cells and CD39^+^ Treg cells [33]. Furthermore, plasma-derived EVs from TNBC patients can directly induce intrinsic apoptosis in CD4^+^ and CD8^+^ T cells while sparing B and NK cells by carrying multiple immunosuppressive ligands, including PD-L1, PD-1, Fas, FasL, TRAIL, CTLA-4, and TGF-B1 [34]. Mechanistically, these EVs enter the T cells and trigger mitochondrial stress, resulting in profound intrinsic apoptosis [34]. Furthermore, TNBC-derived EVs carrying a defined combination of miR-185-5p, miR-652-5p, and miR-1246 can reprogram normal fibroblasts into cancer-associated fibroblasts (CAFs) through PATZ1 downregulation. This, in turn, enhances cancer epithelial cell viability, migration, and invasion in patient-derived BC organoid models [35].

EVs released under hypoxic stress reprogram multiple TME compartments simultaneously. These EVs drive monocytes toward an M2-like macrophage phenotype, suppress macrophage phagocytosis, protect tumor epithelial and endothelial cell integrity, and promote CAF activation [36]. Collectively, these effects generate a pro-survival, pro-angiogenic CAF-enriched TME that reinforces immune evasion and tumor aggressiveness [36]. Furthermore, circulating tumor endothelial cell-derived EVs have been shown to act as potent systemic immunosuppressive mediators that accelerate lung metastasis and angiogenesis while driving broad expansion of Ly6G^+^/CD11b^+^ myeloid populations across the lung, spleen, and bone marrow [37]. These TDEV-exposed immune cells also exhibit upregulated expression of PD-L1, PD-1, iNOS, LAG3, and CTLA4, and ROS/NOS, and impaired T cell cytotoxic function [37].

### 2.4. EV-Driven Metastatic and Pre-Metastatic Niche (PMN) Formation

EVs released from TNBC cells have potent metastatic reprogramming capacity. Specifically, TNBC stem cells (CSCs) and differentiated cancer cells (DCCs) were shown to secrete distinct EV populations carrying divergent bioactive cargo with distinct stromal dependencies [38]. Notably, DCC-derived EVs support CSC maintenance, whereas CSC-secreted EVs drive the emergence of cancer-associated myofibroblasts, contributing to endothelial remodeling, invasion and transformation of healthy lung tissue into a receptive premetastatic niche, ultimately facilitating lung metastasis [38]. Furthermore, CD81 has been identified as a critical EV-associated tetraspanin that cooperates with CD44 to regulate TNBC stemness and lung metastasis [39]. Consistent with this niche-conditioning role, TNBC-derived EVs can directly reprogram normal lung epithelial cells by enhancing proliferation and migration [40].

Hypoxic conditions further amplify tumor invasiveness by generating a vesicle population with enhanced pro-invasive properties characterized by increased protein abundance and diversity with enrichment in pathways related to vesicle-mediated transport, RNA metabolism, DNA replication, and cell cycle progression [41]. Collectively, hypoxia-driven EVs act as potent mediators of metastatic reprogramming by increasing MMP2 and MMP9 secretion, modulating integrin β1 and β3 expression and surface availability, reshaping adhesion dynamics that facilitate invasion, and amplifying proteolytic and catabolic programs, all of which reinforce a TME permissive to invasion [41].

Additionally, TNBC-derived EVs present in patient serum have been shown to transfer the oncogenic lncRNA small nucleolar RNA host gene 4 (SNHG4), thereby enhancing tumor cell growth, survival, and migration, accelerating tumor progression [42]. Mechanistically, SNHG4 upregulates the nuclear export factor XPO5, thereby amplifying XPO5-dependent RNA processing pathways that support oncogenic signaling [42]. TNBC-derived EVs can also directly induce platelet aggregation, vascular protection, immune evasion, and efficient metastasis through urokinase plasminogen activator surface receptor (uPAR) and platelet-derived growth factor receptor beta (PDGFRβ) signaling [27]. Autophagy status can further modulate these metastatic phenotypes through EV-delivered metformin, altering angiogenic signaling, endothelial migration, and metastatic behavior in TNBC cells [43]. Complementing these mechanisms, TNBC-derived EVs enriched in nucleoside diphosphate kinase B (NDPK-B) also activate purinergic signaling in vascular endothelium, driving endothelial migration, barrier disruption, pulmonary vascular leakiness, and pre-metastatic niche (PMN) formation in the lungs [44]. Similarly, plasma-derived EVs enriched in reticulon-4 (RTN4) also potently accelerate metastatic progression by activating NF-κB signaling to drive EMT, PD-L1 upregulation, and CD8^+^ T cell exclusion, thereby promoting lung metastasis and exhibiting synergy with PD-L1-targeted therapy [45]. Similarly, TNBC-derived EVs can aberrantly package the stress-responsive transcription factor NUPR1, which is transferred to endothelial and stromal cells to activate pro-metastatic transcriptional programs, thereby enhancing EV biogenesis and metastatic progression [46].

Additionally, EV-associated cancer-testis antigen SPANXB1 is markedly upregulated in TNBC and functions as a direct promoter of metastatic progression by enhancing migration, invasion, and EMT [47]. Adding another layer to EV-mediated organotropism, TNBC cells were shown to release disproportionately high levels of large EVs. Their biodistribution and lung tropism are governed by specific surface membrane proteins, including CD9, CD44, and SLC29A1 [25]. Tumor-intrinsic regulators such as annexin A2 (AnxA2) further shape the protein composition of TNBC-derived EVs, enriching them with cytoskeletal and adhesion-related cargo and promoting lung-specific PMN formation [25]. Similarly, TNBC-derived EVs enriched in the metastasis-promoting protein Kindlin-2 (K2) were shown to restore nuclear K2 signaling in K2-deficient cancer cells and activate fibroblasts into α-SMA^+^/FAPβ^+^ CAFs [48]. The secretome of these CAFs further amplifies tumor invasion, thereby reinforcing both tumor-intrinsic and stromal pathways of PMN formation [48]. Supporting the central role of endothelial conditioning in metastatic seeding, circulating galectin-3 enhances tumor-endothelium adhesion by driving glycolysis-dependent upregulation of ICAM-1, thereby strengthening vascular enrichment. Additionally, TNBC-derived EVs program macrophages into a pro-metastatic phenotype through a tightly regulated CCL5 expression critical for cytokine release and subsequent invasion [26]. Co-injection of EV-educated macrophages with TNBC cells significantly increases lung metastasis, macrophage accumulation, and Treg infiltration, demonstrating that EV-educated macrophages can actively promote metastasis [26].

Beyond the lungs, TNBC-derived EVs also orchestrate a liver PMN formation through an inflammatory cascade. Specifically, EV-induced TNF-alpha expression upregulates endothelial CX3CL1, recruiting CXCR1^+^ macrophages that express MMP9 and remodel the hepatic microenvironment to facilitate cancer cell invasion [49]. Furthermore, TNBC-derived EVs prime the hepatic PMN by inducing endothelial-to-mesenchymal transition in liver, disrupting vascular barrier integrity, and upregulating fibronectin through EV-associated TGF-β1 signaling [50].

In bone metastasis, TNBC cells secrete ICAM1-enriched EVs that directly induce CD8^+^ T cells exhaustion, suppress cytokine production and activation, and thereby promote bone metastasis [51]. Tumor-derived EVs further promote bone metastasis by enhancing osteoclast formation and function via miRNA-mediated signaling [52]. Specifically, in isolated tumor-derived EVs, osteoclast marker genes, including Acp5, Ctsk, Mmp9, and Nfatc1, are upregulated, in response to these EVs, correlating with enhanced bone metastasis [52]. Additionally, EV-associated miR-21 in BC also contributes to osteolytic PMN formation by inactivating osteoblasts [53].

TNBC-derived EVs induce macrophage polarization and lymph node metastasis [54]. These EVs enhance macrophage migration and induce polarization towards the protumoral M2 phenotype, characterized by exosomal marker CD206 upregulation and inducible nitric oxide synthetase (NOS2) downregulation [54]. Intravenous injection of these EVs promoted primary TNBC tumor growth and subsequent axillary lymph node metastasis [54].

TNBC-derived EVs further promote tumor growth and enhance metastatic potential through biomechanical signaling pathways. Specifically, they activate Hippo signaling pathways, decrease cellular stiffness, reorganize the cytoskeleton and focal adhesions, decondense chromatin, and activate YAP-dependent mechano-transduction, thereby increasing cellular deformability and enhancing the invasive competence required for metastasis [55]. In addition to these biomechanical alterations, highly metastatic TNBC cells selectively load their EVs with unsaturated diacylglycerols (DGs), revealing a lipid-driven mechanism that supports PMN formation by activating PKD/PKCµ signaling in endothelial cells and stimulating pro-angiogenic remodeling [56].

Environmental and systemic metabolic factors also shape EV-mediated metastatic signaling. Bisphenol A (BPA) exposure potentiates metastatic capability of TNBC cells through EV-mediated increase in migration, invasion, and MMP9 secretion [57]. Moreover, systemic metabolic states shape the signaling content of circulating EVs towards a more invasive and survival-advantaged phenotype. EVs derived from obese or insulin-resistant models have been shown to exacerbate TNBC progression and metastasis, linking metabolic dysfunction to EV signaling [58]. Plasma-derived EVs from women with metabolic disorders or obesity actively reprogram TNBC cells toward a more aggressive phenotype, characterized by pro-invasive effects such as elevated MMP2 and MMP9 activity, reduced p53 expression, and a shift in Bax/Bcl-2 balance toward an anti-apoptotic state [59].

While TNBC-derived EVs extensively remodel distant organs to create pre-metastatic niches, metastatic sites can reciprocally influence tumor behavior. Brain organoid-derived EVs have been shown to profoundly reprogram TNBC cells to adapt to the brain microenvironment by inducing EMT, upregulating immunosuppressive PD-L1, increasing the expression of neural markers such as GFAP, enhancing release of miR-194-5p and miR-205-5p, and increasing secretion of inflammatory and pro-invasive cytokines such as MCP-1, IL-6, and IL-8 [60,61].

### 2.5. EV-Mediated Chemoresistance and Phenotypic Plasticity

EVs released from TNBC cells promote chemoresistance and can directly reprogram non-tumorigenic breast epithelial cells toward more aggressive and therapy-resistant phenotypes [62]. TNBC-derived EVs markedly increase the proliferation of breast epithelial cells and confer their resistance to docetaxel and doxorubicin (DOX), demonstrating that EV cargo can impose malignant traits even on genetically normal recipient cells [62]. Furthermore, transcriptomic and miRNA profiling has revealed extensive rewiring of 138 genes and 70 miRNAs, with enrichment in PI3K/AKT, MAPK, and HIF1A signaling pathways central to survival, metabolic adaptation, and stress tolerance in TNBC [62]. Additionally, paclitaxel-resistant TNBC cells upregulate the transcription factor CEBPD, which directly binds to promoter of VAMP3, a vesicle-associated membrane protein involved in intracellular trafficking [63]. Beyond chemoresistance, this pathway also increases extracellular PD-L1 levels, leading to EV-mediated CD8^+^ T cell exhaustion and overall immune evasion [63]. Similarly, with the CEBPD/VAMP3 axis, chemotherapy itself can generate a second wave of immunosuppressive vesicles in which apoptotic TNBC cells release CXCL1-enriched EVs that drive PD-L1^+^ tumor-associated macrophage (TAM) expansion through EED-mediated transcriptional upregulation and fuel metastatic progression [64]. Furthermore, DOX increases the secretion of PTX3-enriched EVs that prime the lung PTM formation and accelerate growth of metastatic tumors [65]. Extending these EV-mediated drug resistance mechanism, PARP inhibitor-resistant TNBC cells secrete EVs enriched in miR-181a that inhibits STING and IFN-γ signaling, causing resistance to both PARP inhibitors and platinum agents [66].

Tyrosine phosphorylation of lactate dehydrogenase A (LDHA) is essential for the selective loading and secretion of circSEPT9 into EVs, enabling its transfer to recipient TNBC cells and driving DOX resistance [67]. Similarly, TNBC cells secrete β-catenin-enriched EVs that drive radioresistance and CSC expansion [68]. Clinical transcriptomic analyses reveal post-radiotherapeutic upregulation of CTNNB1, MYC, and CD44 in TNBC, underscoring EV-associated β-catenin as a therapy-induced driver of CSC plasticity and radioresistance in TNBC [68].

Collectively, these studies show the EVs participate in nearly all stages of TNBC progression. However, much of the current mechanistic evidence is derived from cell culture systems and animal models, highlighting the risk of bias and the need for validation in larger patient cohorts to establish the clinical relevance of these pathways.

## 3. EVs as TNBC Biomarkers

TNBC is often diagnosed at late stages, leading to reduced patient survival, especially in the high-risk populations associated with age, ethnicity, genetic mutations, and family history. Notably, the odds of TNBC diagnosis in women under the age of 40 are around 1.5 times greater than in those aged 60–69 years [69].

### 3.1. EV Isolation, Characterization, and Molecular Profiling Approaches

EVs are increasingly recognized as promising diagnostic tools, as they carry biologically active molecules such as DNA, mRNA, metabolites, and noncoding RNAs (ncRNAs) [70]. They are present in a wide range of biological fluids such as blood, saliva, urine, nasal secretions, breast milk, and cerebrospinal fluid (CSF), making them attractive candidates for liquid biopsy. To fully harness their diagnostic potential, each of these sample sources requires optimized EV isolation and analytical procedures [71]. Plasma-derived EVs are commonly isolated by differential ultracentrifugation, which separates vesicles by sequential high-speed spins but may introduce protein contamination. Hence, additional purification steps are often required to enable accurate vesicular characteristics, including lipid and protein profiles, with those of their possible cellular sources. Precise quantification and source identification further require complementary approaches, such as vesicle flow analysis combined with flow cytometry, using cell type-specific antibodies [71]. Flow cytometry is widely used to validate the presence of canonical EV surface markers, including CD9, CD63, and CD81 [72]. Other widely used isolation methods include density gradient centrifugation, which improves purity by layering vesicles based on buoyant density, and size exclusion chromatography, which gently separates EVs from soluble proteins while preserving vesicle integrity [73]. Although traditional ultracentrifugation is the standard technique, available commercial kits such as ExoQuick, miRCURY and TIER are often preferred in clinical settings because they require less time and smaller sample volume [73]. More recently, specialized enrichment strategies have been developed to selectively isolate tumor-derived EVs from the overwhelming background of normal plasma vesicles [74,75]. These approaches significantly increase the relative abundance of cancer-associated EVs and improve the detectability of tumor-specific proteins, nucleic acids, and surface markers, thereby enhancing the sensitivity and clinical utility of EV-based liquid biopsy platforms.

Once isolated, EVs are typically characterized using high-resolution scanning electron microscopy (HR-SEM) for morphology, high-resolution transmission electron microscopy (HR-TEM) for surface ultrastructure, nanoparticle tracking analysis (NTA) for size distribution, flow cytometry for molecular marker detection, and proteomic or genomic profiling to define molecular cargo [74,75]. Other characterization techniques include Western blotting for the detection of specific marker proteins such as CD63, digital droplet PCR for identification of cancer-related mutations within EVs, nanopore technology for charge-based differentiation, surface plasmon resonance (SPR) used as an efficient and highly sensitive alternative to ELISA, Raman spectroscopy for molecular profiling, and Fourier-transform infrared spectroscopy (FTIR) for chemical group determination [76].

A recently developed Sub-ExoProfile microfluidic platform enables the multiplex immunoisolation and proteomic profiling of CD81, EpCAM, and HER2-positive EV subpopulations, facilitating accurate diagnosis of BC subtypes, including TNBC, using minimal plasma volumes [12]. This further supports the use of marker-defined EV populations for BC molecular subtyping.

### 3.2. EVs as Diagnostic Biomarkers

#### 3.2.1. AI-Driven and Computational Approaches for TNBC Diagnosis

With the emerging importance of artificial intelligence (AI) and machine learning, it is crucial to integrating EV-based TNBC diagnosis with multi-omics, and computational analytics has become increasingly important for improving diagnostic accuracy [76]. For example, integration of single-cell RNA sequencing data with bulk transcriptomic data derived from TNBC patients using computational methods such as LASSO regression has identified EV-related genes consistently expressed in tumor cells and derived EVs [77]. This has enabled stratification of patients with distinct survival potential. Machine learning models have further validated the predictive strength across multiple cohorts, showing the importance of genes including FOSB, CYC1, HMGB2, KPNA2, GBP1, PLA2G5, and EIF4EBP1 in TNBC identification and progression [77,78]. A recently developed rapid, low-cost, and minimally invasive diagnostic approach can organize EVs into reproducible nanoscale spatial signatures for subsequent AI analysis to accurately distinguish EVs derived from different TNBC subtypes and non-cancer controls [79].

Computational oncological approaches in other malignancies further illustrate the promise of these strategies. For example, an example model developed for pancreatic ductal adenocarcinoma (PDAC) was developed to analyze a panel of biomarkers, including EV-derived mRNA/miRNA such as hsa-miR-6803, hsa-miR-1180, hsa-miR-4728, hsa-miR-1915, and hsa-miR-940, in addition to circulating DNA, KRAS mutations, and CA19-9 protein levels. This model achieved an 84% accuracy rate in detecting PDAC before metastasis [80,81]. These findings demonstrate the effectiveness of machine learning in outperforming conventional imaging approaches and highlight the potential as a preoperative screening tool for identifying patients eligible for surgical intervention [82]. Another important application is the integration of deep learning with surface-enhanced Raman spectroscopy (SERS) data derived from circulating EVs for the diagnosis of very early stage lung cancer achieving an accuracy of approximately 91% [83]. This approach utilizes a residual neural network (ResNet) architecture and once again proves the potential of deep learning to enhance early cancer detection, a strategy that could be readily expanded to early diagnosis of TNBC [83].

TNBC cells also exhibit distinct glycosylation remodeling, producing EVs enriched with sialylated and fucosylated glycans on their surface [84]. This molecular feature enables the use of advanced techniques such as the lectin-based thermophoretic assay (EVLET), which profiles the glycan signatures of circulating EVs in a sensitive, and minimally invasive strategy that combines vibrating membrane filtration (VMF) with thermophoretic amplification [84]. Importantly, this platform allows direct quantification of glycan signatures from microliter-scale plasma samples in less than 100 min, enabling discrimination of TNBC patients from healthy individuals and those with benign cancer conditions [84]. Sequential labeling approaches enable precise, multitarget analysis of EV surface proteins at the single-vesicle level, and by performing multiple rounds of antibody labeling, imaging, and signal removal. This method overcomes the limitations of conventional immunostaining and identifies distinct protein signatures and marker patterns that distinguish cancer-derived EVs with high sensitivity, thereby offering a promising platform for liquid biopsy-based cancer diagnostics [85]. Digital assays further enhance diagnostic sensitivity by achieving extremely low limits of detection (LOD), reaching sub-femtomolar sensitivity, and offering robust biomarker identification within clinically relevant ranges [86]. In these assays, individual biomolecules are compartmentalized into discrete complexes according to Poisson statistics, after which signals are amplified and detected as binary positive/negative events allowing precise quantification of vesicles and their molecular cargo [86].

#### 3.2.2. Multi-Omics in Biomarker Discovery

Multi-omic approaches, which integrate genomics, transcriptomics, proteomics, and metabolomics, have emerged as powerful strategies for EV-based TNBC diagnosis and can be further strengthened through computational analysis. Among these, serum proteomics provides highly informative protein expression profiling [86]. Through efficient protein extraction and solubilization combined with two-dimensional electrophoresis (2DE) using fluorescence stains such as Sypro dyes and Cy-dyes, followed by mass spectrometry-based EV profiling could be completed, offering enhanced resolution and proteome coverage across thousands of proteins can be achieved [87]. This offers a comprehensive, highly sensitive, and accurate platform for the early detection of TNBC. Proteomic profiling has also revealed distinct protein signatures capable of differentiating healthy individuals, patients with benign breast disease, and those with TNBC [88]. Among these groups, TNBC exhibits the most divergent proteomic landscape, enriched in proteins associated with tumor progression, immune modulation, and metastatic behavior. These findings support the utility of EV-associated proteins, such as HISTH2A, which can serve as minimally invasive plasma-based biomarkers for TNBC liquid biopsy applications [88]. In addition, lipidomic and metabolomic profiling of EVs provides valuable insight into the roles of EVs in cancer development, progression, and intercellular communication mechanisms, further expanding their utility in cancer screening [76,89]. Building on this, another metabolomic profiling study has explored the metabolites in sEVs derived from the MDA-MB-231 TNBC cell line and the MCF10A non-tumorigenic breast epithelial cell line [90]. Through dual validation and the use of high-resolution liquid chromatography–mass spectrometry (LC-MS) and multivariate computational modeling, two metabolomic biomarkers unique to TNBC-derived sEVs, LysoPC 0:0/22:6 and N-acetyl-L-phenylalanine, were identified. These candidate biomarkers further underscore the value of integrating metabolomics with EV biology for improved TNBC detection [90]. Machine learning further enhances multi-omic biomarker discovery by employing ensembles of computational algorithms that integrate multiple methods, including linear discriminant analysis, logistic regression, naïve Bayes, and support vector machines to yield more reliable predictions in the diagnosis of liquid and serum biopsies. Similarly, deep learning utilizes algorithms into layered artificial neural networks capable of autonomous decision-making and complex pattern recognition, further improving the sensitivity and robustness of EV-based diagnostics [76].

#### 3.2.3. Integration of AI and Machine Learning with Multi-Omic Profiling for TNBC Diagnosis

The integration of AI and machine learning with multi-omic profiling offers a transformative opportunity for EV-based diagnosis of TNBC and other malignancies. By integrating diverse molecular layers including proteins, nucleic acids, lipids, glycans, and metabolites, multi-omic approaches can capture the full complexity of EV cargo thereby enabling earlier detection and more accurate disease staging. Although heterogeneity and lack of methodological standardization remain major challenges, the convergence of multi-omics and AI-driven analytics represents a next generation liquid biopsy platform with strong potential to overcome these challenges and advance precision oncology across diverse cancer types. Another particularly promising component of EV-based diagnostics is mitochondrial DNA (mtDNA). Recent studies have identified approximately 168 somatic nonsynonymous mutations distributed across various coding (73%) as well as noncoding (27%) regions [89], with 11 hotspot mtDNA mutations in 19 to 38% of the patients [89]. Enrichment and sequencing analysis of mtDNA from serum-derived EVs isolated from nine TNBC cases led to identification of 45 mtDNA mutations encompassing coding and non-coding regions with at least one EV-associated mutation corresponding to that present in the primary tumor. These mutations include both homoplasmic and heteroplasmic variants, particularly within the genes encoding the oxidative phosphorylation machinery [89]. Additionally, TNBC patients exhibit significantly higher burdens of EV-associated mtDNA mutations than healthy controls, suggesting that EV-associated mtDNA may serve as a highly sensitive, non-invasive biomarker for TNBC detection and disease monitoring [89]. Another innovative diagnostic strategy involves Vcpp2319, a cell-penetrating peptide with anti-metastatic properties that interacts with EVs released by metastatic BC cells and exploits these vesicles to traverse a human blood–brain barrier (BBB) model [91]. These EV-bound complexes demonstrated significantly enhanced translocation compared with the free peptide, indicating that metastatic EVs may function as biological shuttles that facilitate peptide delivery into the brain compartment [91].

Even though AI-discovered signatures demonstrated promising diagnostic and prognostic value in retrospective transcriptomic datasets, their prospective clinical validation in TNBC patients is still lacking and could represent a major future direction. Additionally, multi-omic approaches are superior to single biomarker analyses in terms of comprehensiveness; however, their integration into routine clinical workflows can be limited by cost, technical complexity, and the need for standardized protocols.

Hence, we can say that AI potential in biomarker discovery mainly lies in integrating complex multi-omic data into clinically actionable tools that will need validation, rather than evaluating EV biomarkers.

### 3.3. EVs as Prognostic Biomarkers in TNBC

While diagnostic biomarkers allow the identification of TNBC, prognostic biomarkers provide critical insight into disease behaviors and clinical outcomes. TNBC generally experiences poorer outcomes compared with other BC subtypes due to its aggressive clinical behavior, lack of well-defined molecular targets, significant high risk of recurrence, and elevated metastatic potential [92]. Among the most important prognostic biomarkers are EVs, which carry a diverse array of molecular cargo including DNA, miRNAs, proteins, and metabolites that closely reflect tumor progression and disease dynamics. However, not all proposed prognostic biomarkers have reached the same level of evidence, and many remain at the discovery stage pending validation in independent clinical cohorts.

#### 3.3.1. lncRNAs as Prognostic Biomarkers

EV-derived long non-coding RNAs (lncRNAs) play crucial roles in TNBC progression and metastasis, holding substantial prognostic value. EVs derived from hypoxic tumor-associated macrophages (TAMs) were isolated and subjected to RNA sequencing, revealing 73 differentially expressed lncRNAs, including 32 upregulated and 41 downregulated transcripts [93]. Among these, EV-derived MIR210HG was markedly upregulated in TNBC TME and showed a strong correlation with disease progression, reaching peak expression in patients with stage IV disease [93]. Treatment of TNBC cells with TAM-derived EVs overexpressing MIR210HG resulted in efficient uptake and increased MIR210HG levels in recipient cells, supporting its role in EV-mediated communication between hypoxic TAMs and TNBC cells [93]. Functionally, EV-associated MIR210HG promotes M2-polarization and metastatic aggressiveness by upregulating neighboring genes such as RASSF7 [93]. Another clinically relevant EV-derived lncRNA is XIST. This form of XIST is significantly upregulated in nearly 50% of recurrent TNBC patients and its low levels is highly correlated with good patient survival [94].

#### 3.3.2. miRNA as Prognostic Biomarker

EV-associated miRNAs represent highly informative prognostic biomarkers linked to TME remodeling and therapeutic resistance [95]. RNA sequencing of TNBC-derived EV cargo has revealed that miR-185-5p, miR-652-5p, and miR-1246 synergistically activate fibroblasts toward a pro-migratory functional phenotype [96]. This is particularly relevant given the established role of CAF in supporting invasion and metastasis [11]. In addition, TNBC-derived miRNAs contribute to immune evasion and chemoresistance and may serve as prognostic biomarkers. For example, EV-associated miR-20a-5p inhibits CD8^+^ T cell function by significantly reducing levels of IFNγ, TNF-α, perforin, and granzyme B, all of which are critical mediators of the anti-tumor immune response [11]. Furthermore, miR-20a-5p directly targets and suppresses NPAT, a gene essential for rapid cell cycle progression and proliferation [11]. EV-associated miRNAs are also strongly linked to chemoresistance. High-throughput screening of EV-derived miRNA from patients’ blood samples revealed eight miRNAs expressed differentially between patients with no response and complete response to neoadjuvant chemotherapy (NAC) [97]. Heatmap analysis demonstrated that non-responsive patients exhibit downregulation of miR-185, miR-4283, miR-5008 and miR-3613 and upregulation of miR-1302, miR-4715 and miR-3144 [97]. Gene set enrichment analysis further revealed that pathways associated with immunity control were most affected, suggesting that immune dysfunction contributes significantly to poor NAC response in this TNBC patient subpopulation [97].

#### 3.3.3. Proteins and Other Prognostic Biomarkers in TNBC

Recent work profiling drug-resistant proteins within EVs revealed that markers such as the transmembrane proteins MDR1, MRP1, and BCRP are significantly enriched in patients who are non-responsive to NAC [98]. These EV-derived drug resistance signatures accurately predicted NAC non-response with high sensitivity and specificity [98]. Further supporting the prognostic versatility of EVs and representing the opposite side of the spectrum, it has been shown that annexin A2 (AnxA2) protein and mRNA loaded within small EVs serve as a combined predictive biomarkers for responsiveness to chemotherapy in aggressive TNBC cases [99]. High levels of AnxA2 are associated with advanced disease stage and consistently observed in patients who responded to NAC, while non-responders were shown to exhibit low levels of AnxA2. This observation may provide a minimally invasive indicator for treatment sensitivity [99]. Additionally, the overexpression of integrin beta 4 (ITGB4) in TNBC cells transfers ITGB4 to CAFs via EVs [100]. A known function of CAFs in cancer progression is the Warburg effect, which is a process that involves the production of high-energy metabolites via aerobic glycolysis [100]. The Warburg effect was found to be elevated in CAFs receiving ITGB4, supporting its pivotal role in cancer progression [100]. Additionally, ITGB4-dependent glycolysis was also associated with mitochondrial fission and clearance, enhancing the energy balance and stress adaptation in TNBC cells [100]. Another major regulator of the TME is caveolin-1 (Cav1), which favors cell elongation in three-dimensional cultures and promotes Rho- and force-dependent contraction, matrix alignment, and cancer cell migration, invasion and metastasis [101]. Additionally, proteomic profiling of circulating EVs has identified recurrence in TNBC patients by quantifying EV-associated proteins enriched during relapse, such as extracellular matrix protein 1 (ECM1), hemoglobin subunit alpha 1 (HBA1), and prenylcysteine oxidase 1 (PCYOX1), etc. [72]. Other proteins identified that are associated with oncogenic growth and proliferative signaling pathways include PDXN, GGT5, and SERPINE1 [102]. Collectively, through multi-omic technologies, EVs can offer minimally invasive means of detecting and monitoring the immune molecular states, predicting chemotherapy sensitivity, guiding immunotherapy use, and developing multifunctional platforms and gene risk models for precision TNBC management [84]. Recent work has expanded the prognostic utility of EVs even further by examining their glycan landscapes. Through the development of an EV lipidomic and thermophoresis (EVLET) system, which is based on thermophoretic profiling, it was demonstrated that EVs from TNBC patients exhibit distinct glycosylation signatures, particularly altered sialylation and fucosylation, that differentiate them from healthy individuals and correlate with tumor burden and treatment response [84]. The EV glycan signature was found to represent a weighted sum of 3 lectins, ConA, WGA, and RCA I. Together, these findings underscore that EVs represent a highly integrative platform capable of capturing the molecular, metabolic, and immunologic heterogeneity of TNBC, which enhances prognostic precision, treatment stratification, and disease monitoring [84].

Furthermore, identifying whole molecular clusters and gene-risk models can help determine chemo-immunotherapy vulnerability rather than relying solely on individual EV biomarkers [103]. Through integrated transcriptomics, proteomics, and EV profiling in mouse models, four main clusters of TNBC heterogeneity were identified [103]. These clusters include DC-enriched (strong proliferative activity and interferon signaling), macrophage enriched (EMT features and mesenchymal programs), T cell-enriched (heterogeneous proliferative states), and neutrophil-enriched (elevated OXPHOS metabolism and tumor necrosis). Key EV-associated diagnostic and prognostic biomarkers in TNBC are summarized in Table 1.

## 4. Therapeutic Applications of EVs in TNBC

TNBC is known to be a difficult target for traditional chemotherapy and immunotherapy due to its high heterogeneity, formidable stromal barriers, and lack of target receptors such as HER-2, PR, and ER [104]. Additionally, chemotherapeutic drugs such as paclitaxel have been shown to promote chemoresistance, enhanced aggressiveness, and immune evasion [105]. As previously discussed, EVs actively contribute to TNBC disease progression and therapeutic resistance, which supports their potential as therapeutic targets. In addition to their established role as minimally invasive diagnostic and prognostic biomarkers, EVs are increasingly being explored as therapeutic delivery systems for proteins, lipids, nucleic acids, immune modulators, and nanotechnology-based agents [106]. This is due to their beneficial properties, including their small size, natural biocompatibility, intrinsic targeting ability, efficient cellular uptake, and engineerable flexibility [107]. Although EV-mediated therapies against some solid tumors have advanced to clinical trials and those against TNBC demonstrate compelling mechanistic and therapeutic potential, EV-based interventions for TNBC remain entirely preclinical, with limited TNBC-specific EV therapeutics currently in Phase I/II clinical evaluation [108].

**Figure 2:**
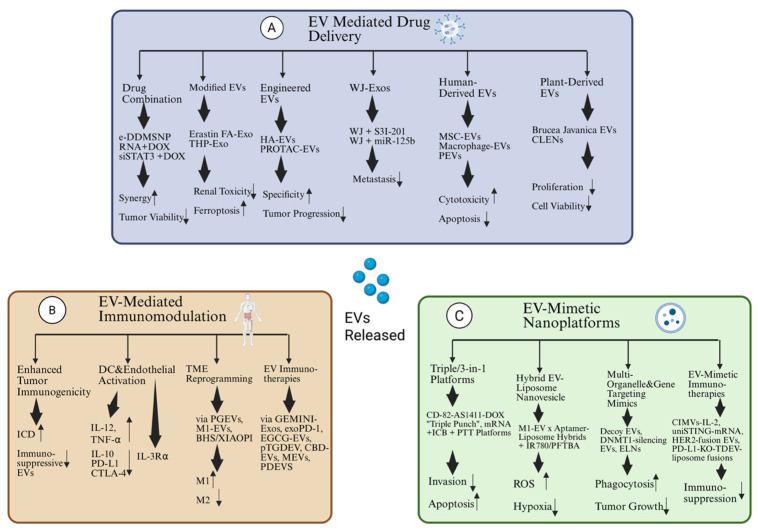
**EV-Based Therapeutic Strategies.** (**A**) EV-mediated drug delivery platforms that enhance tumor targeting, reduce toxicity, and suppress proliferation and metastasis; (**B**) EV-mediated immunomodulation that enhances tumor immunogenicity, promotes dendritic and endothelial activation, and supports EV-based immunotherapies; (**C**) EV-mimetic nanoplatforms that reduce invasion, alleviate hypoxia, and reverse immunosuppression. Abbreviations: Apt-Lips, aptamer-conjugated liposomes; BHS, Baohuoside I; CBD, cannabidiol; CD, cluster of differentiation; CIMVs, cytochalasin B-induced membrane vesicles; CLENs, citrus-derived extracellular nanovesicles; CTLA-4, cytotoxic T-lymphocyte-associated protein 4; DC, dendritic cell; DIM, 3,3′-diindolylmethane; DOX, doxorubicin; e-DDMSNP, EV-encapsulated DIM-doxorubicin mesoporous silica nanoparticle; EGCG, epigallocatechin gallate; ELNs, EV-lipid hybrid nanoparticles; EMT, epithelial-to-mesenchymal transition; ERK, extracellular signal-regulated kinase; EV, extracellular vesicle; FA-Exo, folate-modified exosome; GEMINI-Exos, genetically engineered multifunctional immunomodulatory exosomes; ICD, immunogenic cell death; IL-3Rα, interleukin-3 receptor alpha; IL-10, interleukin-10; IL-12, interleukin-12; IL-6, interleukin-6; IL-8, interleukin-8; M1, pro-inflammatory macrophage subtype; M2, immunosuppressive macrophage subtype; MEVs, milk-derived extracellular vesicles; MSC, mesenchymal stem cell; PD-1, programmed cell death protein 1; PD-L1, programmed death-ligand 1; PDEVs, plant-derived extracellular vesicles; PFTBA, perfluorotributylamine; PGEVs, Platycodon grandiflorum-derived EVs; PI3K, phosphoinositide 3-kinase; PLGA, poly(lactic-co-glycolic acid); poly I:C, polyinosinic-polycytidylic acid; PROTAC, proteolysis-targeting chimera; PTGDEV, pyroptosis-triggering genetically engineered vesicle; STAT3, signal transducer and activator of transcription 3; THP, pirarubicin; TLR3, toll-like receptor 3; TNF-α, tumor necrosis factor alpha; WJ-Exo, Wharton’s Jelly-derived exosome.

Figure 2 summarizes EV-based therapeutic strategies that are currently being studied in the context of TNBC. As shown in Figure 2A, EVs can be engineered as drug delivery vehicles to enhance their targeting, specificity and therapeutic efficacy. EVs have also been explored for their role in immunomodulation and thus enhancing antitumor immunity and immunotherapy response (Figure 2B). Figure 2C, however, highlights EV mimetic nanoplatforms that are developed recently for TNBC.

### 4.1. EVs as Drug Delivery Vehicles

EVs can function as drug delivery vehicles to target TNBC cells [106]. The use of EVs as nanocarriers ensures biocompatibility, efficient cellular uptake, selective and effective tumor targeting and minimal side effects [107,109]. Notably, co-delivery of 3,3′-diindolylmethane (DIM), a heterocyclic and bioactive compound with anticancer effects, and doxorubicin (DOX), a chemotherapeutic agent that was shown to induce EMT in cancer stem cells (CSCs) and enhance tumor aggressiveness, using mesoporous silica nanoparticles encapsulated within EVs (e-DDMSNP), produced synergistic inhibition of TNBC cell viability [5]. e-DDMSNPs can penetrate the tumor masses, improving drug delivery and efficacy [5]. Similarly, the dual-delivery platform incorporating RNA nanoparticles and chemotherapeutic agents within EVs improves cargo encapsulation and stability [110]. Engineered EVs co-loaded with RNA nanoparticles and chemotherapeutic agents show enhanced uptake by TNBC cells and induces synergistic cytotoxicity at significantly reduced drug doses compared with other free drugs or single-agent EV formulations [110]. Additionally, TDEVs have also been engineered to co-deliver DOX and siSTAT3, which suppresses STAT3 signaling, induces immunogenic cell death, and reverses the immunosuppressive TME, resulting in enhanced dendritic cell maturation, increased M1 macrophage polarization, and elevated CD4^+^ and CD8^+^ T cell infiltration in tumor tissues [111].

Similarly, other treatment agents such as erastin and pirarubucin (THP) are known to induce ferroptotic cell death in TNBC cells and direct tumor cell death through DNA synthesis interference, respectively. However, their clinical utility is limited by poor water solubility, significant renal and cardiac toxicity, and limited distribution to tumors [112]. To overcome these limitations, folate-modified EVs loaded with erastin were developed [112]. This formulation demonstrated markedly stronger cytotoxicity and ferroptosis induction compared to free erastin and unmodified EVs loaded with erastin [112]. Similarly, tumor-derived EVs (TEVs) were engineered to deliver THP [113] and evaluated as a synergistic chemo-immunotherapeutic platform for TNBC. Extending this growing body of evidence supporting EVs as versatile and targetable drug-delivery platforms, hyaluronic acid-engineered milk-derived EVs have been developed to selectively target CD44-overexpressing TNBC cells [114].

Functionalization with high-molecular-weight hyaluronic acid markedly enhanced CD44-mediated uptake and intracellular delivery, resulting in significantly improved targeting specificity toward TNBC cells while minimizing off-target internalization [114]. Furthermore, proteolysis-targeting chimera (PROTAC)-loaded EVs were developed to overcome the poor stability and limited tumor penetration that restrict PROTAC efficacy in TNBC [115]. Upon using a microfluidic droplet-based electroporation system, PROTAC YX968 was efficiently loaded into EVs while maintaining vesicle integrity and achieving substantially higher loading efficiency than conventional methods [115]. EV-mediated delivery also increases the intracellular degradation of HDAC3 and HDAC8, epigenetic regulators implicated in tumor progression, in TNBC cells, resulting in stronger suppression of tumor growth compared to free PROTAC.

Furthermore, EVs derived from Wharton’s jelly mesenchymal stem cells (WJ-Exo) have been engineered as nanocarriers and loaded with S3I-201, a STAT3 inhibitor [116]. WJ-Exo-mediated delivery of S3I-201, suppressed STAT3, reduced cancer cell migratory capacity and induced significantly higher levels of late apoptosis in TNBC compared to free S3I-201, largely through the upregulation of pro-apoptotic genes such as Bax and caspase-3 [116]. Importantly, EV-encapsulation enhanced the *in vivo* potency of S3I-201, demonstrating improved therapeutic efficacy against TNBC [116]. Another WJ-Exo mediated therapy included the loading and delivery of miR-125b, suppressing HIF1α-driven proliferation, EMT, angiogenesis, and metastasis while reprogramming tumor cells to release secondary anti-tumor EVs [117]. Additionally, MSC-derived EVs loaded with miR-3143, miR-424-5p, miR-218, and the lncRNA 7SK and delivered to TNBC cells produced a marked increase in apoptosis, accompanied by significant suppression of cell proliferation, migration, and EMT [118,119,120]. Macrophage-derived EVs have also emerged as effective drug carriers of doxorubicin and paclitaxel due to their intrinsic tumor-homing capacity and immunological compatibility [121]. Drug-loaded EVs exhibited enhanced uptake by TNBC cells, leading to increased cytotoxicity, elevated apoptosis, and significant suppression of proliferation and migration [121]. Similarly, platelet-derived EVs (PEVs) efficiently loaded with doxorubicin were shown to be effective drug-delivery carriers exhibiting controlled drug release, enhanced cytotoxicity against TNBC cells, and increased intracellular drug accumulation [122].

Plant-derived exosome-like nanoparticles (PELNs) extracted from the Brucea javanica fruits contain miRNAs that exhibit anti-angiogenic and anti-tumor activity by suppressing proliferation, metastasis, and enhancing the apoptosis of TNBC cells [104]. Other PELNs such as citrus-derived extracellular nanovesicles (CLENs) were shown to get efficiently internalized by TNBC cells and produce dose and time-dependent reduction in cell viability [123]. CLENs also markedly suppressed TNBC cell migration and invasion through downregulation of phosphorylated PI3K, AKT, and ERK [123]. These findings further highlight the therapeutic promise of EV-mediated RNA restoration strategy in reprogramming TNBC cells toward a less aggressive phenotype.

### 4.2. EVs in Immune Modulatory Applications and Therapeutic Response

EVs are used in immune modulatory applications. Engineered photosensitive mesenchymal stem cell (MSC)-derived EVs co-loaded with the photosensitizer Ce6 and the EV-secretion inhibitor GW4869 (Ce-GW4869/sEVs) have been developed [124]. These EVs selectively accumulate in tumors and enhance photodynamic therapy by promoting immunogenic tumor cell death [124]. Concurrently, GW4869 suppresses TNBC-derived sEV secretion, reducing the release of immunosuppressive vesicles [124]. This dual action improves tumor immunogenicity, promoted immune activation, and alleviated the inhibitory TME [124].

Traditional immunotherapeutic techniques, such as dendritic cell (DC) vaccinations, are often unsatisfactory in TNBC treatment due to the poor immunogenicity, an immunosuppressive TME, and limitations in DC maturation and activation strategy. Therefore, use of TNBC-derived EVs has been shown to enhance the maturation and immunogenicity of monocyte-derived DCs (moDCs) by upregulating IL-12 and TNF-α cytokines and downregulating immune checkpoints that inhibit DC maturation, such as IL-10, PD-L1, and CTLA-4, thereby enhancing DC-mediated anti-tumor activity [125]. Similarly, blocking IL-3R-alpha on tumor-derived endothelial cells reprograms their EV microRNA cargo, especially through loss of miR-24-3p, leading to upregulation of SPRY2 and subsequent apoptosis, inhibition of EMT, and suppression of metastasis [29]. Additionally, platycodon grandiflorum-derived EVs (PGEVs) exhibit anti-tumor activity in TNBC by simultaneously reprogramming the TME and influencing systemic host responses [126]. PGEVs also promoted M1 polarization of TAMs, and enhanced systemic immune responses through altered gut microbiota composition [126]. Furthermore, M1-derived EVs were combined with PLGA nanoparticles carrying a TLR3 agonist (poly I:C) to produce a potent immunomodulatory therapy that reverses tumor immune suppression and reduces TNBC metastasis, as evidenced by increased CD163^+^ M2-like to CD68^+^ M1 polarization of TAMs [127]. Similarly, Baohuoside I (BHS, a CXCR4 inhibitor) and XIAOPI (an herbal medicine) formula therapies have been shown to reverse EV-mediated immunosuppressive signaling by reducing CXCL1 enrichment in chemotherapy-induced EVs released from apoptotic TNBC cells and suppressing PD-L1-dependent M2 polarization, thereby restoring chemosensitivity to paclitaxel [24,105]. To further explore the potential of EVs in immunotherapy, EVs engineered to express four distinct proteins on their surfaces, aCD3- aEGFR-PD-1-OX40L GEMINI-Exos, exhibit potent activity in directing, activating, and modulating T cell-mediated immunity against EGFR-positive TNBC. This successful generation of multifunctional EVs through genetic engineering represents a significant advance in targeted immunotherapy [128].

Programmed cell death protein 1 (PD-1) is an inhibitory immune checkpoint receptor expressed on cytotoxic T cells and tumor-infiltrating lymphocytes (TILs). Upon binding to its ligand (PD-L1) on tumor cells, it mediates T cell exhaustion and inhibits the T cell-mediated anti-tumor immunity. In this context, EVs contribute to immune modulation through the transfer of PD-1. Interestingly, EV-associated PD-1 (exo-PD-1) enhances T cell-mediated cytotoxicity, by binding tumor cell PD-L1 and inducing its internalization via clathrin-mediated endocytosis [129]. Collectively, these effects suppress tumor growth and enhance overall therapeutic response [129].

Additionally, EGCG-derived EVs (EGCG-EVs) suppress the pro-tumoral effects of TNBC-derived EV signaling on adipose-derived MSCs (hADMSCs). They suppress AKT and GSK-3β signaling pathways, reduce expression of inflammatory mediators such as CCL2, and prevent senescence by downregulating p21 and β-galactosidase [130].

In another approach, potent pyroptosis-driven immunological conversion in TNBC has been achieved using engineered EVs [131]. Co-encapsulation of gasdermin E N-terminal GSDME-N mRNA and discoidal domain receptor 1 (DDR1) mRNA has been utilized to trigger pyroptosis and suppress immune exclusion, respectively [131]. This dual-function construct was also functionalized with an anti-CD156 antibody to achieve tumor-specific delivery, yielding pTGDEV, which markedly inhibited tumor progression and increased patient survival in TNBC mouse models [131]. Also, pTGDEV also reduced myeloid-derived suppressor cells and increased DC, CD4^+^, and CD8^+^ T cell infiltration, effectively converting the TME from immunosuppressive to immunostimulatory [131].

Regarding chemotherapy response modulation, cannabidiol-loaded EVs (CBD-EVs), bovine milk-derived EVs (MEVs), and plant-derived EV (PDEVs) can increase chemosensitivity to DOX through different methods. MSC-EVs, exhibited high encapsulation efficiency, stable physicochemical properties, and sustained CBD release under physiological and tumor-like pH conditions [132]. Functionally, CBD EVs markedly reduced tumor burden, decreased expression of inflammation and metastasis-associated proteins, and increased expression of pro-apoptotic markers [132]. Complementing this approach, MEVs also enhance doxorubicin efficacy by targeting metabolic and STAT-driven survival pathways, reducing metabolic fitness, depleting multiple pro-tumoral interferon-inducible proteins and metabolic enzymes, and suppressing STAT family proteins and their downstream oncogenic effectors [133]. Similarly, citrus limon PDEVs synergistically enhance doxorubicin-induced cytotoxicity through modulating metabolic and stress-response pathways, resulting in increased apoptosis and reduced tumor proliferation [134].

### 4.3. EVs Mimetic Nanoplatforms

EV-mimetic nanoplatforms offer a novel and innovative strategy that facilitates TNBC therapeutics. The generation of the “triple punch” cell membrane-derived EV-mimetic nanovesicle system is a prominent example [135]. The combination of an overexpressed metastasis suppressor CD82, an aptamer AS14411 on the nanovesicle, and a chemotherapeutic agent DOX, was proven to inhibit the migration and invasion of TNBC cells [135]. This is particularly important as free DOX was well known for causing substantial off-target toxicity in normal tissues. Using the “triple punch” delivery system can selectively enhance apoptosis in TNBC cells while reducing apoptosis in normal cells [135]. Another “three-in-one- platform” is an EV-based system integrating mRNA therapy, immune checkpoint blockade, and mild photothermal therapy, further illustrating the expanding versatility of EV-derived technologies in TNBC treatment [136]. This enables simultaneous delivery of therapeutic mRNA, disruption of immunosuppressive signaling, and localized photothermal activation, collectively amplifying anti-tumor efficacy [136]. The coordination of these complementary mechanisms within a single EV vehicle enhances tumor cell killing, promotes immune activation, and improves therapeutic precision, underscoring the growing potential of engineered EVs as highly adaptable tools for multimodal TNBC therapy [136].

Similarly, the formation of a hybrid nanovesicle harnessing the advantages of both EVs and liposomes can enhance the functionalities of both systems through bimodal tumor targeting [137]. A hybrid nanoplatform of M1-EVs and AS1411 aptamer conjugated liposomes (A Apt-Lips), loaded with IR780 photosensitizer and perfluorotributylamine (PFTBA) showed high stability and generated high levels of ROS, improving the effectiveness of photodynamic therapy (PDT) [138]. Additionally, measurement of hypoxia-inducible factor-1α (HIF-1α) levels demonstrated that the hybrid can alleviate hypoxia in the TME, thereby reducing the ability of tumor survival, metastasis, and invasion [138].

Moreover, engineered decoy EVs (D-EVs) were developed to overcome the profound heterogeneity of TNBC by enabling sequential cascade-targeting of the cell membrane, cytoplasm, and mitochondria, thereby enhancing tumor tropism and deep intratumoral penetration [139]. This multi-organelle targeting process significantly enhanced intracellular delivery of therapeutics such as TEAD-siRNA and T/MOF, overcoming tumor heterogeneity and inducing robust mitochondrial dysfunction, elevated ROS production, and increased apoptosis through inhibition of aerobic glycolysis and oxidative phosphorylation [139]. Similarly, by engineered DNMT1-silencing EVS, immunosuppressive MDSCs are deleted, the TME is remodeled, and the PD-1 checkpoint blockade is enhanced [140]. Additionally, EV-lipid hybrid nanoparticles (ELNs) used to enhance siRNA delivery and therapeutic efficacy in TNBC cells were shown to target CD24, CD44, and CD47 with efficiencies over 50%, resulting in a robust gene silencing, enhanced macrophage-mediated phagocytosis, and overall tumor inhibition [141].

Another EV-mimetic based therapy is cytochalasin B-induced membrane vesicles (CIMVs) generated from IL-2-overexpresing MSCs. This therapy produces stronger cytotoxic responses than both IL-2 or MSCs alone, reduces proliferation and viability of TNBC cells, decreases tumor growth up to four-fold, and reduces T cell suppression and Treg expansion [142]. Additionally, a universal stimulator of interferon genes (STING) mimic overcomes major limitations of conventional STING agonists by activating IRF3/IFN-I tumor-control pathways independently of endogenous STING expression [143]. In multiple tumor models, including TNBC, lipid nanoparticle-delivered uniSTING-mRNA elicited antitumor immunity by promoting DC maturation and antigen-specific CD8+ T cell responses while inhibiting NF-KB driven tumor-promoting inflammation [143]. These treated tumor cells were found to release EVs enriched with miRNAs that suppressed the immunosuppressive factor Wnt2b and further enhanced DC activation [143]. The combined use of uniSTING-mRNA and anti-Wnt2b antibodies produced robust tumor inhibition and extended survival [143].

A novel technique involved in HER-2 positive EVs were fused with TNBC cells to confer de novo HER2 expression, enabling targeted drug delivery [144]. Specifically, HER-2-grafted TNBC tumors exhibited markedly improved therapeutic responses to the targeted liposomal paclitaxel, suggesting new avenues for EV-based TNBC-targeting strategies [144]. Another innovative strategy utilizes TDEVs as a two-stage chemoimmunotherapy platform where PD-L1-knockout TDEVs fused with doxorubicin-loaded liposomes to achieve high drug encapsulation (~97%), enabling homologous tumor targeting, enhanced immunogenic tumor cell death, reduced tumor aggression, suppressed metastasis, and minimal off-target effects [145].

## 5. Challenges and Future Directions

Despite the rapid progress in EV research, there remain several technical and biological challenges that continue to limit the full clinical integration of EV-based diagnostics and therapeutics in TNBC. A major barrier remains the heterogeneity of EV populations, which vary widely in size, biogenesis, cargo composition, and functional potency [74,75]. This diversity complicates the isolation and characterization of TNBC-EVs against the overwhelming background of vesicles released by normal tissues [74,75]. Research-grade EV isolation often relies on small-volume, low-throughput methods that lack batch consistency [146]. Current isolation methods including ultracentrifugation, size-exclusion chromatography, density gradients, and commercial precipitation kits, each introduces biases in purity, yield, or vesicle integrity, underscoring the need for standardized, reproducible, and scalable protocols suitable for clinical deployment [73]. Clinical-grade production requires GMP-compatible, closed-system workflows such as tangential-flow filtration, high-resolution SEC, and immune-affinity capture [147,148]. Likewise, clinical implementation demands rigorous quality control metrics including cargo stability, sterility testing, potency assays, and orthogonal quantification (NTA, IFC, ddPCR, proteomics), methods rarely applied in preclinical studies [148]. Without harmonized standards for isolation, quantification, and release criteria, EV-based liquid biopsies and therapeutics cannot meet regulatory expectations, making manufacturing and analytical validation major obstacles to real-world translation. Notably, many foundational TNBC-EV studies were conducted prior to MISEV 2023 and therefore lack the orthogonal characterization and purity controls discussed. This complicates interpretation of earlier “exosome-specific” findings and limits clinical reproducibility.

Biological variability further complicates translation. EV cargo is dynamically shaped by microenvironmental cues such as hypoxia, ECM stiffness, metabolic stress, and therapeutic exposure. Even micropatterned surface topography has been shown to modulate EV biogenesis, with aligned micro-tracks significantly increasing EV output in TNBC cells [143]. These context-dependent shifts raise important questions about how to define “diagnostic” or “prognostic” EV signatures across diverse patient states and treatment timelines. Adding to these context-dependent sources of variability, TNBC itself is a molecularly heterogenous disease comprising various molecular subtypes, with each subtype displaying unique ontologies, age at diagnosis, grade, disease progression, and response to chemotherapy [145]. These subtypes include basal-like, mesenchymal, immunomodulatory, and luminal-androgen receptor (LAR) subtypes. These various subtypes have also been shown to alter EV cargo composition, with basal-like tumors releasing EVs enriched in proliferation and DNA damage-associated cargo and mesenchymal tumors producing EVs carrying EMT-related proteins and miRNAs [7,21,149]. Although still unexplored, subtype-resolved EV profiling may enable EV-based liquid biopsies to function as non-invasive surrogates for TNBC subtype classification, improving precision oncology and patient stratification.

Building on the differences in EV cargo within TNBC molecular subtypes, EV profiles also diverge substantially across the major breast cancer subtypes. These subtype-specific signatures limit the clinical generalizability of TNBC-derived EV markers to ER+/PR+ and HER2+ tumors. TNBC-derived EVs are typically enriched in pro-invasive and EMT-associated proteins such as vimentin and annexin 2, oncogenic miRNAs, and metastasis-promoting factors [18,25,150]. In contrast, ER+/PR+ tumors tend to release EVs enriched in hormone regulated transcripts and metabolic regulators, consistent with a less aggressive phenotype [95]. HER2+ tumors frequently secrete EVs containing HER2 proteins and associated signaling molecules such as GRB7 [151,152]. These subtype-specific EV signatures highlight the biological divergence between breast cancer subtypes and underscore the need for subtype-resolved EV biomarker development in breast cancer, particularly TNBC.

Prognostic EV biomarkers face additional hurdles including small sample sizes, limiting statistical power and generalizability [76]. Larger, multi-center cohorts are essential to validate EV-based prognostic signatures and ensure their robustness across populations. Moreover, multi-omic integration and AI-driven classification offer powerful solutions but introduce their own challenges, including data harmonization, cohort standardization, and the need for large, diverse datasets to avoid overfitting [76]. While these computational approaches can help address EV heterogeneity and enhance diagnostic precision, their clinical adoption will require rigorous validation, regulatory frameworks, and cross-platform compatibility.

Therapeutically, EV-based delivery systems show enormous promise but face practical constraints such as cargo loading. Although innovative strategies such as microfluidic droplet-based electroporation for PROTAC loading, dual-delivery platforms combining RNA nanoparticles with chemotherapeutics, and the rapamycin-inducible FKBP-FRB system for dose-controlled loading have significantly improved encapsulation efficiency and vesicle integrity, these technologies must be optimized for large-scale manufacturing [110,115,153]. Ensuring consistent biodistribution, minimizing off-target effects, and establishing long-term safety profiles are essential steps before EV-based therapeutics can enter routine clinical use.

Despite these challenges, the field is advancing rapidly. Emerging technologies in microfluidics, high resolution vesicle profiling, glycan mapping, and machine learning are poised to address many of these limitations. As methodological standardization improves and mechanistic insights deepen, EV-centered diagnostics and therapeutics hold strong promise for transforming TNBC detection, monitoring, and treatment.

## 6. Conclusions

EVs have emerged as central orchestrators of TNBC biology, functioning not merely as passive biomarkers but also as active drivers of tumor progression, metastatic niche formation, immune evasion, and therapeutic resistance. Their ability to transport oncogenic proteins, lipids, nucleic acids, and metabolites enables TNBC cells to remodel the TME, reprogram stromal and immune compartments, and condition distant organs for metastasis. These same properties allow EVs to act as powerful tools for early detection, prognostic stratification, and therapeutic intervention. Advances in multi-omic profiling, high-resolution vesicle characterization, and AI-driven analytics have accelerated the discovery of EV-associated signatures that predict treatment response. On the other hand, engineered EVs and EV-mimetic nanoplatforms have demonstrated remarkable versatility as delivery vehicles for RNA and chemotherapeutics and immune modulators. Collectively, these developments underscore the transformative potential of EV-centered technologies in reshaping TNBC management. By integrating EV-based diagnostics, prognostic modeling, and therapeutic engineering, the field is moving toward a precision oncology framework in which EVs serve as both molecular readouts and actionable therapeutic platforms. Continued mechanistic insight into EV biogenesis, cargo selection, and intercellular communication will be essential for translating these advances into clinically deployable strategies.

Nonetheless, important challenges remain, including EV heterogeneity, methodological variability in EV isolation and characterization, and, most importantly, the limited clinical validation of many proposed biomarkers and therapeutic platforms, that are in most cases are still in early preclinical stages. Addressing these challenges using standardized methods and clinical studies is essential for understanding the full potential of EV based precision oncology in TNBC.

## Figures and Tables

**Table 1:** EV-associated biomarkers in TNBC.

Biomarker Category	Biomarkers	EV Source/Biological Material	Clinical Utility	Refs.
EV-Surface Markers (Diagnostic)	CD9, CD63. CD81. EpCAM, HER2	Plasma EVs, Sub-ExoProfile microfluidic platform	EV identification; TNBC subtype classification; liquid biopsy enrichment	[12,72]
EV DNA/ mtDNA Biomarkers	168 somatic mtDNA mutations, 11 hotspot mutations, OXPHOS-related mutations	Serum-derived EVs	Early detection; TNBC vs healthy discrimination; monitoring mutation burden	[89,103]
EV-RNA Biomarkers (in general)	mRNA, ncRNA	Blood, saliva, urine, CSF, breast milk	Broad diagnostic potential; multi-fluid liquid biopsy	[93]
Glycan-Based Biomarkers	Sialylated glycans, fucosylated glycans; EVLET signature (ConA, WGA, RCA-I)	Circulating EVs	TNBC detection; subtype discrimination; correlates with tumor burden	[84]
Metabolomic Biomarkers	LysoPC 0:0/22:6; N-acetyl-L-Phenylalanine	sEVs from TNBC vs non-cancer cells	TNBC-specific metabolic signature; early detection	[90]
Proteomic Biomarkers (Diagnostic)	HISTH2A, ECM1, HBA1, PCYOX1, PDXN, GGT5, SERPINE1	Circulating EVs	Distinguish TNBC from benign/healthy; recurrence detection	[88]
AI-Derived Gene Signatures	FOSB, CYC1, HMGB2, KPNA2, GBP1, PLA2G5, EIF4EBP1	EV-related gene sets (scRNA-seq + bulk RNA-seq)	Risk stratification; TNBC identification; survival prediction	[77,78]
AI-Patterning Biomarkers	Freeze–thaw nanoscale EV spatial signatures	Plasma EVs	Rapid, low-cost subtype-specific EV diagnosis	[79]
Cross-Cancer AI Biomarkers (Relevant Models)	hsa-miR-6803, miR-1180, miR-4728, miR-1915, miR-940	EVs from PDAC/lung cancer (model systems)	Demonstrate ML potential for EV-based early detection; applicable to TNBC	[80,81]
Spectroscopy-Based Biomarkers	SERS EV spectral signatures (ResNet-classified)	Circulating EVs	Stage I cancer detection; high accuracy; adaptable to TNBC	[83]
Proteomic Multi-Omics Clusters	DC-enriched, macrophage-enriched, T cell-enriched, neutrophil-enriched clusters	Integrated EV + transcriptomic + proteomic profiling	Predict chemo-immunotherapy vulnerability; TNBC heterogeneity mapping	[103]
lncRNA Prognostic Biomarkers	MIR210HG (hypoxic TAM-EVs), XIST (exo-XIST), SNHG4	TAM-EVs; serum EVs	Predict metastasis, recurrence, tumor burden, NAC response	[93]
miRNA Prognostic Biomarkers (TME/Metastasis)	miR-185-5p, miR-652-5p, miR-1246	TNBC-derived EVs	CAF activation; pro-migratory TME remodeling	[96]
miRNA Prognostic Biomarkers (Immune Evasion)	miR-20a-5p	TNBC-EVs	CD8+ T cell suppression; immunotherapy resistance	[11]
miRNA Predictors of NAC Response	**Lower in non-responders:** miR-185, miR-4283, miR-5008, miR-3613; **Higher in non-responders:** miR-1302, miR-4715, miR-3144	Patient plasma EVs	Predict neoadjuvant chemotherapy response	[97]
Drug-Resistance Protein Biomarkers	MDR1, MRP1, BCRP	EVs from NAC non-responders	Predict chemotherapy resistance; high sensitivity/specificity	[98]
Predictive Protein Biomarkers (Treatment Sensitivity)	Annexin A2 (AnxA2) protein + mRNA	Small EVs	Predict NAC responsiveness; high AnxA2 = better response	[99]
Metabolic/CAF-Reprogramming Biomarkers	ITGB4 (drives Warburg effect), Cav1 (matrix alignment, migration)	EVs from TNBC cells	Predict stromal remodeling, invasiveness, metabolic rewiring	[100]
Recurrence-Associated EV Proteins	ECM1, HBA1, PCYOX1, PDXN, GGT5, SERPINE1	Circulating EVs	Recurrence prediction; relapse monitoring	[72]
Specialized Biomarker Systems	Vcpp2319 peptide–EV complex	Metastatic EVs crossing BBB	Diagnostic delivery tool; brain-penetrant EV biomarker	[91]

Abbreviations: AI, artificial intelligence; AnxA2, annexin A2; BBB, blood–brain barrier; BCRP, breast cancer resistance protein; CAF, cancer-associated fibroblast; CD, cluster of differentiation; CSF, cerebrospinal fluid; DC, dendritic cell; DNA, deoxyribonucleic acid; ECM1, extracellular matrix protein 1; EIF4EBP1, eukaryotic translation initiation factor 4E binding protein 1; EpCAM, epithelial cell adhesion molecule; EV, extracellular vesicle; EVLET, extracellular vesicle lipidomic and thermophoretic profiling system; FOSB, FosB proto-oncogene; AP-1 transcription factor subunit; GBP1, guanylate binding protein 1; GGT5, gamma-glutamyltransferase 5; HER2, human epidermal growth factor receptor 2; HBA1, hemoglobin subunit alpha 1; HISTH2A, histone cluster 2 H2A family member; HMGB2, high mobility group box 2; ITGB4, integrin beta 4; KPNA2, karyopherin subunit alpha 2; lncRNA, long noncoding ribonucleic acid; LysoPC, lysophosphatidylcholine; MDR1, multidrug resistance protein 1; miRNA, micro ribonucleic acid; MRP1, multidrug resistance-associated protein 1; mtDNA, mitochondrial deoxyribonucleic acid; NAC, neoadjuvant chemotherapy; ncRNA, noncoding ribonucleic acid; OXPHOS, oxidative phosphorylation; PCYOX1, prenylcysteine oxidase 1; PDAC, pancreatic ductal adenocarcinoma; PDXN, peroxidasin; PLA2G5, phospholipase A2 group V; ResNet, residual neural network; RNA, ribonucleic acid; SERS, surface-enhanced Raman spectroscopy; SERPINE1, serpin family E member 1; sEV, small extracellular vesicle; SNHG4, small nucleolar RNA host gene 4; TAM, tumor-associated macrophage; TME, tumor microenvironment; TNBC, triple-negative breast cancer; Vcpp2319, vascular cell-penetrating peptide 2319; WGA, wheat germ agglutinin.

## Data Availability

No new data were created or analyzed in this study. Data sharing is not applicable.

## Abbreviations

AA	arachidonic acid
AI	artificial intelligence
Apt-Lips	aptamer-conjugated liposomes
BC	breast cancer
BBB	blood–brain barrier
BHS	Baohuoside I
CAA	cancer-associated adipocyte
CAF	cancer-associated fibroblast
CBD	cannabidiol
CD	cluster of differentiation
CIMVs	cytochalasin B-induced membrane vesicles
CLENs	citrus-derived extracellular nanovesicles
CSC	cancer stem cell
CSF	cerebrospinal fluid
DCC	differentiated cancer cell
DCs	dendritic cells
DDR1	discoidin domain receptor 1
DIM	3,3′-diindolylmethane
D-EVs	decoy extracellular vesicles
ECM	extracellular matrix
EGCG	epigallocatechin gallate
EGFR	epidermal growth factor receptor
ELNs	EV-lipid hybrid nanoparticles
EMT	Epithelial-to-mesenchymal transition
ER	estrogen receptor
EV	extracellular vesicle
EVLET	extracellular vesicle lipidomic and thermophoresis
EVsCSC	CSC-derived EVs
EVsDCC	DCC-derived EVs
EVh	hypoxic extracellular vesicle
FA-exo	folate-modified exosome
FTIR	Fourier-transform infrared spectroscopy
GEMINI-Exos	genetically engineered multifunctional immunomodulatory exosomes
GW4869	neutral sphingomyelinase inhibitor
HA	hyaluronic acid
HER2	human epidermal growth factor receptor 2
HIF-1α	hypoxia-inducible factor 1 alpha
HR-SEM	high-resolution scanning electron microscopy
HR-TEM	high-resolution transmission electron microscopy
ISEV	International Society for Extracellular Vesicles
LC-MS	liquid chromatography–mass spectrometry
LOD	limit of detection
MEVs	milk-derived extracellular vesicles
ML	machine learning
MVB	multivesicular body
MSC	mesenchymal stem cell
NAC	neoadjuvant chemotherapy
NTA	nanoparticle tracking analysis
OXPHOS	oxidative phosphorylation
PCYOX1	prenylcysteine oxidase 1
PDEVs	plant-derived extracellular vesicles
PEVs	platelet-derived extracellular vesicles
PFTBA	perfluorotributylamine
PGEVs	Platycodon grandiflorum-derived EVs
PDT	photodynamic therapy
PDAC	pancreatic ductal adenocarcinoma
PMN	pre-metastatic niche
PR	progesterone receptor
PROTAC	proteolysis-targeting chimera
PTM	pre-tumoral microenvironment
sEV	small extracellular vesicle
SERS	surface-enhanced Raman spectroscopy
SPR	surface plasmon resonance
STAT	signal transducer and activator of transcription
TAM	tumor-associated macrophage
TDEVs	tumor-derived extracellular vesicles
TEVs	tumor-derived EVs
TGF-β	transforming growth factor beta
THP	pirarubicin
TIS	therapy-induced senescence
TLR3	toll-like receptor 3
TME	tumor microenvironment
TNBC	triple-negative breast cancer
Treg	regulatory T cell
UC	ultracentrifugation
VMF	vibrating membrane filtration
WJ-Exo	Wharton’s Jelly-derived exosomes
YAP	Yes-associated protein
